# Breathing new life into an old target: pulmonary disease drugs for Parkinson’s disease therapy

**DOI:** 10.1186/s13073-017-0483-4

**Published:** 2017-10-19

**Authors:** Hisham Abdelmotilib, Andrew B. West

**Affiliations:** Center for Neurodegeneration and Experimental Therapeutics, Department of Neurology, The University of Alabama at Birmingham, Birmingham, AL 35294 USA

**Keywords:** Epigenetic modification, Histone acetylation, Lewy body disease, Neuroinflammation, Neuroprotection, Parkinson’s disease, SNCA

## Abstract

Increases in α-synuclein protein expression are suspected to increase the risk of the development of Parkinson’s disease (PD). A recent study has demonstrated that β2-adrenergic receptor (β2AR) agonists decrease histone acetylation in the α-synuclein gene and suppress transcription. Coupled with the anti-inflammatory effects that are associated with β2AR activation, this two-pronged attack holds promise for PD treatment and the development of new therapeutic approaches for this disease.

## Parkinson’s disease and the role of α-synuclein

Human genetic studies have shown that increases in the expression of *SNCA*, the gene encoding the α-synuclein protein, may increase the risk of the development of Parkinson’s disease (PD). In rare familial PD cases, copy number variants that result in multiplications of the *SNCA* gene cause an aggressive early-onset PD phenotype [1]. In idiopathic PD cases that lack mutations in the *SNCA* gene, genome-wide association studies have identified PD-associated promoter variants and variants in the 5’ and 3’ untranslated regions (UTRs) that may lead to increased *SNCA* expression [2]. The α-synuclein protein forms inclusions known as Lewy bodies and neurites that spread throughout much of the brain in PD-affected individuals. Intense research efforts have focused either on strategies to reduce the propensity of α-synuclein to aggregate, or on reducing the expression of α-synuclein. Here, we consider the genetic and epigenetic mechanisms that are involved in α-synuclein gene regulation and how these might inform future PD interventions.

## Identification of a new target in Parkinson’s disease

In a recent study, Mittal et al. provided evidence that β2-adrenergic receptor (β2AR) agonists, which are the most common medications used for respiratory diseases, are associated with reduced PD risk in the Norwegian population [3]. Drugs that activate β2ARs (agonists) mimic the effects of endogenous catecholamines, including norepinephrine, epinephrine, and dopamine, and have effects on smooth muscle. β2AR agonists dilate bronchial passages and are used in asthma treatment and can relax uterine muscles and so are used in treating preterm labor. β2AR blockers, such as propranolol, antagonize epinephrine and norepinephrine and have broad utility in treating cardiovascular disease. In general, both β2AR blockers and long-acting agonists, as well as some short-acting agonists, penetrate the blood–brain barrier. In the study by Mittal et al., β2AR agonists were found to decrease *SNCA* expression in neurons in different experimental models. In defining the underlying mechanism, the authors showed that β2AR blockers increased, whereas agonists decreased, histone 3 lysine 27 acetylation in the α-synuclein promoter. Epigenetic mechanisms such as histone acetylation at the *SNCA* promoter have been shown to regulate gene expression by loosening chromatin and by improving the accessibility of chromatin for transcription factor binding [4]. *SNCA* mRNA levels could be reduced by an impressive ~ 30% in neurons exposed to salbutamol (also known as albuterol), metaproterenol, and clenbuterol, which are all β2AR agonists commonly used for the treatment of asthma. Based on the genetic risk imposed by increases in α-synuclein expression, a sustained 30% decrease in α-synuclein expression in the brain may have a substantial effect on PD susceptibility.

A high degree of overlap in the expression profiles in cells and tissues between β2AR and α-synuclein might provide confidence that this axis can be broadly targeted to decrease transcription factor binding in the *SNCA* promoter and thus the corresponding gene transcription. Surveys of carefully curated expression databases of human cells and tissues have revealed a wide distribution of β2AR in the body with notably high expression in immune cells [5]. By contrast, α-synuclein is primarily expressed in the brain, and a recent study using advanced genetic-sorting technology to isolate different mouse brain cell types for deep mRNA sequencing showed little or no expression of the β2AR gene (*ADBR2*) in cortical neurons compared with very high expression in microglial and endothelial populations that lacked *SNCA* [6].

The very high *β2AR* expression in microglia has not previously gone unnoticed, and β2AR agonists have demonstrated some efficacy in reducing neuroinflammation and neurodegeneration in multiple models of neurodegeneration [5]. Therefore, Mittal et al. have identified an exciting second purpose for β2AR agonists in reducing *SNCA* expression in some neurons, presumably those that express β2AR. Importantly, α-synuclein protein strongly activates pro-inflammatory responses in the brain. Therefore, the two expected therapeutic activities associated with β2AR agonists are not mutually exclusive. Even if β2AR agonists fail to act on *SNCA* in some neurons due to a lack of β2AR expression, there may still be therapeutic gain through a broad dampening of microglial activation caused by abnormal α-synuclein expression.

## Lessons learned from epidemiological studies

Another remarkable observation from Mittal et al. was that Norwegians who were using the short-acting β2AR agonist salbutamol were less likely to develop PD [3]. In subjects who had asthma and chronic obstructive pulmonary disease, the reduction in PD risk was dependent on the duration of salbutamol use. With the longest duration of use (at least 6 months of treatment), 25 subjects developed PD compared with the ~ 43 subjects who would be expected to develop PD. Shorter salbutamol use of 2 months had no effect on PD risk. In turn, more than 1 year of treatment with the β2AR blocker propranolol for cardiac disease resulted in an increased risk of PD, with 41 subjects developing PD versus the ~ 17 subjects who would be expected to develop PD. Shorter durations of propranolol use had no effect on PD risk.

The Norwegian population has a similar PD incidence to that of other well-studied populations. Outside of Norway, there may be several well-powered cohorts available to confirm or refute the associations found by Mittal et al. For example, the Danish National Registry and the National Parkinson’s Patient Register in Denmark have been useful for identifying inverse associations between smoking and PD. The General Practice Research Database in the United Kingdom has been used to decipher the association between anti-inflammatory drugs and PD risk. However, there are two areas of caution for future studies. First, propranolol is commonly used to treat essential tremor, which is a known and potent risk factor for PD, whereas salbutamol may be used to treat lung disease that is caused by smoking, which is a known and potent protective factor against PD risk. In a Taiwanese prospective cohort study of ~ 10,000 asthmatic patients treated with various β2AR agonists, the incidence of PD was increased and correlated with the severity of asthma [7]. This association was not reported by Mittal et al.; in their study, PD risk was unchanged in patients with asthma who were treated with inhaled corticosteroids after adjusting for salbutamol use and smoking. It will not be straightforward to correct for both the known and the unknown biases that influence PD risk, and multiple as yet unknown genetic and environmental factors are likely to drive the directions and strengths of these associations.

## Challenges with translating existing drugs to the clinic

Mittal et al. used intraperitoneal injection of US Food and Drug Administration (FDA)-approved agonists to demonstrate efficacy in blocking *SNCA* promoter acetylation and in reducing *SNCA* expression in the mouse brain. In pre-clinical studies, β2AR agonists have demonstrated efficacy in reducing inflammation and neurodegeneration in cerebral ischemia, traumatic brain injury, and even in tau pathology models, but their positive effects required pretreatment [5]. Epidemiological studies will be needed to clarify the timing of agonist exposure relative to PD diagnosis. Furthermore, pre-clinical studies should evaluate whether treatment paradigms, rather than prevention (pretreatment) paradigms, have effects in models that rely on endogenous α-synuclein for neurodegeneration. Fortunately, such models are now in use in the PD research field [8].

Currently no β2AR agonists have been specifically developed for PD. Repurposing existing drugs may involve compromises in brain penetration, oral availability, half-life, specificity, and safety in elderly populations. However, there is a time delay and resource drain associated with de novo efforts to develop optimized new molecules. The early failure of a less-than-optimal β2AR agonist in efficacy trials may have an industry-wide effect by leading to the discontinuation of programs that aim to bring superior molecules to trial. Regrettably, in PD clinical research, most efficacy trials have ended without any measurable end point due to a lack of knowledge of whether the drug had successfully engaged the desired target or produced the intended effect. When considering data from Mittal et al. and others, there is a clear mechanism of action in the reduction of α-synuclein levels and the possible reduction of neuroinflammation. These effects can be monitored in clinical trials using biomarkers and imaging approaches and incorporated early into rationally conceived development pipelines. One challenge is that α-synuclein levels in cerebral spinal fluid are already suppressed in PD populations [9], potentially reflecting compensatory changes, so early clinical studies should determine whether further reductions in α-synuclein levels are possible. The findings of Mittal et al. breathe new life into an old target and provide hope that disease modification in PD will be possible in the near future.

## Abbreviations

PD: Parkinson’s disease
SNCA: Alpha-synuclein
β2AR: β2-adrenergic receptor
